# Phenotypic characteristics of myelin oligodendrocyte glycoprotein antibody-associated disease in children: a single-center, retrospective study

**DOI:** 10.3389/fneur.2023.1188323

**Published:** 2023-08-29

**Authors:** Yi Hua, Xuke Yan, Liu Liu, Yilong Wang, Lu Xu, Peifang Jiang, Zhefeng Yuan, Feng Gao

**Affiliations:** Department of Neurology, Children’s Hospital, Zhejiang University School of Medicine, National Clinical Research Center for Child Health, Hangzhou, Zhejiang, China

**Keywords:** myelin oligodendrocyte glycoprotein, myelin oligodendrocyte glycoprotein antibody-associated disease, clinical phenotype, diagnosis, treatment

## Abstract

**Objective:**

To analyze the clinical characteristics and follow-up data of children with different clinical phenotypes of myelin oligodendrocyte glycoprotein antibody-associated disease (MOGAD).

**Methods:**

The basic demographic and clinical features, laboratory and imaging examination results, and follow-up data of 74 Chinese children with different phenotypes of MOGAD were retrospectively reviewed and analyzed.

**Results:**

The male-to-female ratio in this cohort was 1:1.39. The clinical phenotypes of MOGAD included acute disseminated encephalomyelitis (ADEM; *n* = 37), encephalitis (*n* = 11), optic neuritis (ON, *n* = 9), neuromyelitis optica spectrum disorder (NMOSD; *n* = 9), transverse myelitis (TM; *n* = 6), leukodystrophy-like manifestations (*n* = 1), and meningitis (*n* = 1). The mean age of disease onset was 86 months. The number of leukocytes in the cerebrospinal fluid of patients with ADEM was significantly higher than that in patients with ON but lower than that in patients with TM (*p* < 0.05). The pathogen detection rate among all patients was 36.5%. Recurrence occurred in 17 patients (23%), with the highest recurrence rate in patients with NMOSD and TM. Patients with recurrence had a significantly higher median age than those without any recurrence (109.00 vs. 82.44 months, *p* < 0.05). The male-to-female ratio in patients with recurrence was 1:4.67, which differed significantly from that at first onset (*p* < 0.05).

**Conclusion:**

The most common clinical phenotypes of MOGAD in this cohort were ADEM and encephalitis. Recurrence of MOGAD may be related to age and sex, with a higher recurrence rate observed in females. These findings provide a basis for further exploration of the characteristics of different MOGAD phenotypes.

## 1. Introduction

Myelin oligodendrocyte glycoprotein (MOG) is a myelin protein that belongs to the immunoglobulin (Ig) superfamily (1). It is exclusively expressed on the surface of myelin sheaths and oligodendrocyte membranes in the central nervous system (CNS) (2). MOG antibody-associated disease (MOGAD) is an idiopathic, rare, immune-mediated inflammatory demyelinating disease of the CNS, which overlaps with other clinical presentations, such as acute disseminated encephalomyelitis (ADEM), neuromyelitis optica spectrum disorder (NMOSD), optic neuritis (ON), and multiple sclerosis (MS) (3). The neuropathological hallmarks of MOGAD consist of MOG-dominant myelin loss, white matter demyelination, intracortical demyelination, etc. (4). Among demyelinating CNS disorders, the relative frequency of MOGAD seems higher in children than in adults (5). It affects both female and male children, with a similar frequency in both genders (6). Recent evidence has suggested that anti-MOG immunoglobulin G (MOG-IgG) may be a pathogenic antibody against MOGAD (7). As a disease with a wide clinical spectrum, the clinical features of MOGAD warrant further investigation. In the present study, we aimed to summarize and analyze the characteristics, treatments, and follow-up data of Chinese children with different clinical phenotypes of MOGAD.

## 2. Materials and methods

### 2.1. Study population

In this retrospective study, children who were diagnosed with MOGAD and treated in the Department of Neurology of Children’s Hospital Affiliated to Zhejiang University School of Medicine between March 2017 and February 2021 were qualified for screening. The inclusion criteria were as follows: (1) aged ≤18 years; (2) diagnosed with MOGAD according to the provisional criteria proposed by the International Pediatric Multiple Sclerosis Study Group Criteria (2012) (8) and the International MOGAD panel (9); (3) presented with symptoms of autoimmune encephalitis or fever; (4) tested positive for serum MOG antibodies by cell-based assay; and (5) with complete medical records. Patients were excluded if they (1) presented with another CNS disease, such as cerebral palsy and neonatal brain injury; (2) also had a systemic immune disease, such as lupus erythematosus; (3) were positive for other CNS demyelinating antibodies, such as anti-aquaporin-4 (AQP4) antibody. The experimental protocols were approved by the local ethics committee (approval no. 2021-IRB-161). The parents or guardians of all children were informed of treatment plans and provided written informed consent before laboratory and imaging examinations.

### 2.2. Data collection

The demographic characteristics and clinical data, including clinical manifestations, laboratory and imaging examination results, treatments, and follow-up data of all patients were collected from the medical records and analyzed retrospectively. All children were tested for serum MOG-IgG using fixed cell-based assay (Euroimmun AG, Germany) with a full-length human MOG as the target antigen and a low positive cut-off value of 1:10. The samples were sent to the V-Medical Laboratory (Hangzhou, China) for analysis. All patients who tested positive for MOG antibodies underwent magnetic resonance imaging (MRI) examinations of the brain and spine, including T1, T2, and fluid-attenuated inversion recovery (FLAIR).

Cerebrospinal fluid (CSF) samples were collected from 71 patients by lumbar puncture. Testing for Epstein–Barr virus (EBV) and herpes simplex virus (HSV) was performed in 55 and 52 patients, respectively. The serum EBV antibody test and *mycoplasma pneumoniae* antibody test were performed on 60 and 58 patients, respectively.

### 2.3. Treatments

All patients received methylprednisolone pulse therapy at a dosage of 10–20 mg/kg/day per day for 3–5 days. Concomitant gamma globulin immunomodulatory therapy at a dosage of 1 g/kg/day was administered to 43 patients for 2 days.

The immunosuppressive therapy regime consisted of azathioprine (2 mg/kg/day, divided into two oral doses), methotrexate (20 mg/kg/day, divided into two oral doses), cyclophosphamide (750 mg/m^2^, administered once every 4 weeks for a total of 4 cycles), and intravenous immunoglobulin (1 g/kg/day, administered for 2 consecutive days).

### 2.4. Statistical analysis

Data were analyzed using SPSS 20.0 software both before and after the stratification of different clinical phenotypes and treatments. The normality of the data distribution was tested using the Kolmogorov–Smirnov test. Continuous variables with a normal distribution were compared using the Student’s *t*-test. Categorical variables were compared using the Chi-square test. The test standard was *p* = 0.05, and *p* < 0.05 was considered statistically significant.

## 3. Results

### 3.1. Basic demographic and clinical characteristics

**Figure 1:**
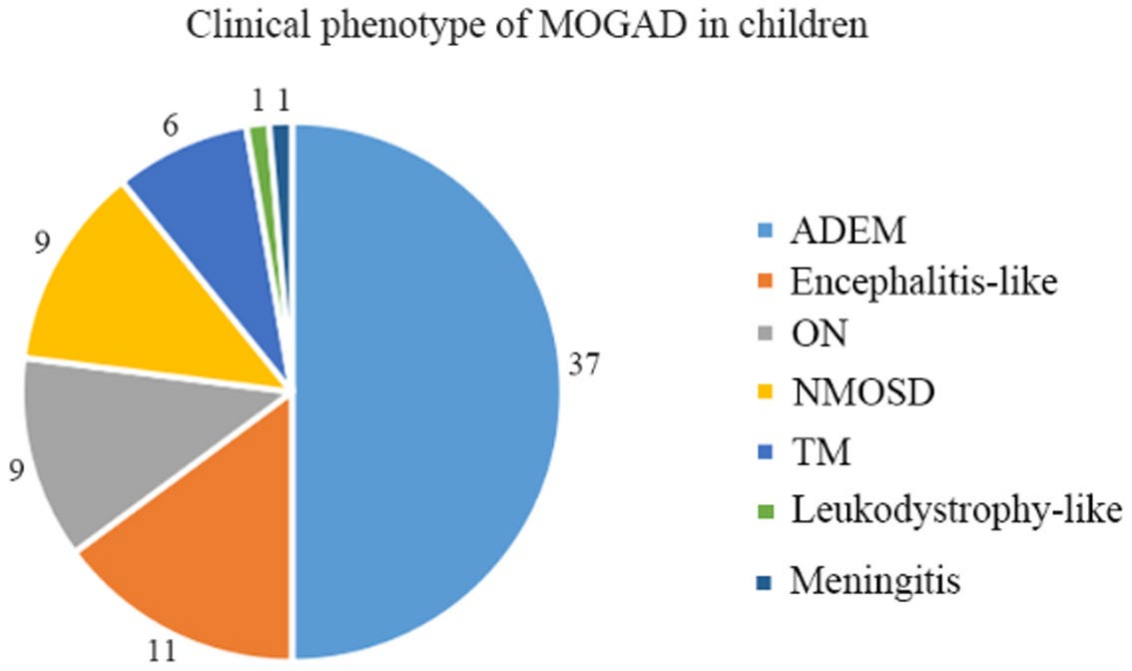
Clinical phenotypes of MOGAD in Chinese children.

A total of 74 children qualified for inclusion in this study, including 31 boys and 43 girls, for a male-to-female ratio of 1:1.39. Different clinical phenotypes were observed in patients with MOGAD, including ADEM (*n* = 37, 50.0%), encephalitis (*n* = 11, 14.9%), ON (*n* = 9, 12.1%), neuromyelitis optica spectrum disorder (NMOSD, *n* = 9, 12.1%), transverse myelitis (TM, *n* = 6, 8.1%), leukodystrophy-like manifestations (*n* = 1, 1.4%), and meningitis (*n* = 1, 1.4%) (Figure 1).

**Figure 2:**
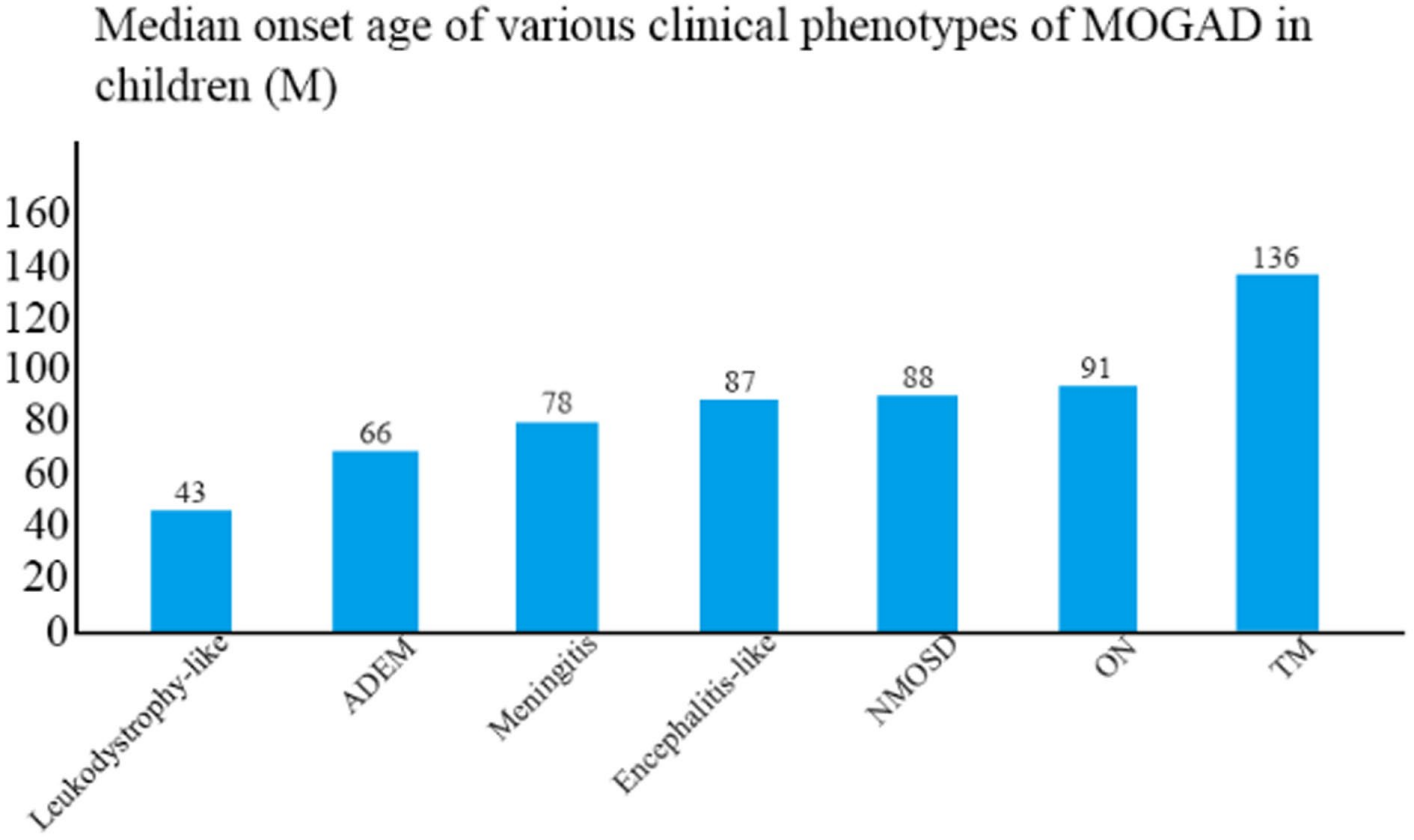
Median age of onset among children with different clinical phenotypes of MOGAD.

The median age of disease onset was 86 months (range, 20–175 months). The clinical phenotype with the lowest mean age of onset was leukodystrophy-like manifestations (43 months), followed by ADEM (66 months) and meningitis (78 months), and the one with the highest mean age of onset was TM (136 months) (Figure 2). The mean age of onset differed significantly between TM and ADEM and between TM and ON (both *p* < 0.05).

**Figure 3:**
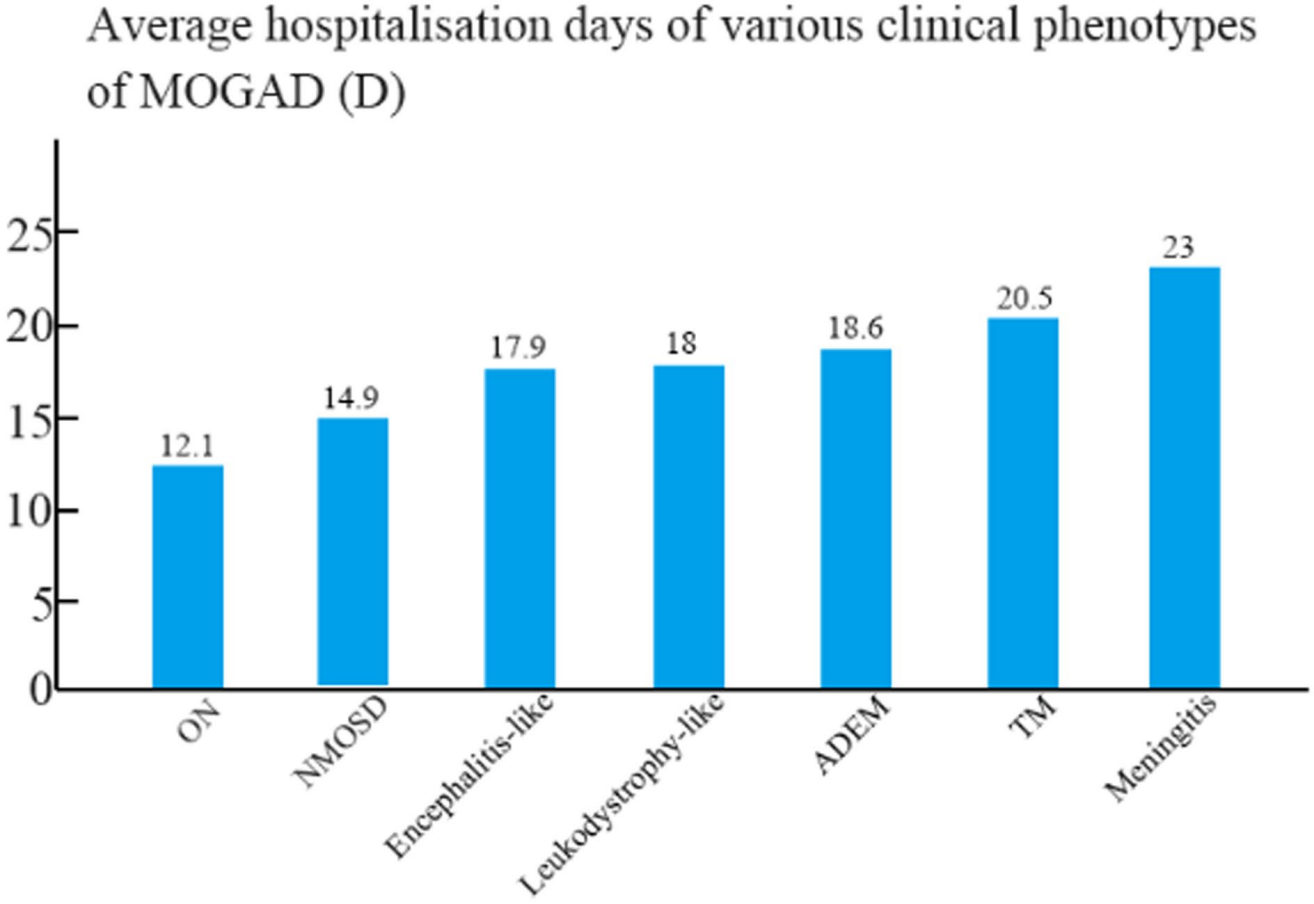
Mean length of hospital stay among children with different clinical phenotypes of MOGAD.

The mean hospital stay was 18.6 days for patients with ADEM, 17.9 days for patients with encephalitis, 12.1 days for patients with ON, 14.9 days for patients with NMOSD, 20.5 days for patients with TM, 18 days for patients with leukodystrophy-like manifestations, and 23 days for patients with meningitis (Figure 3). The mean hospital stay of patients with ON differed significantly from those of patients with ADEM, TM, and encephalitis (*p* < 0.05).

The clinical features observed in the various MOGAD presentations included fever (*n* = 31), headache (*n* = 19), dizziness (*n* = 10), vomiting (*n* = 11), hypersomnia (*n* = 10), seizures (*n* = 14), visual impairment (*n* = 18), ophthalmalgia (*n* = 2), muscle weakness (*n* = 16), dysuria (*n* = 4), constipation (*n* = 2), ataxia (*n* = 5), mood changes (n = 1), facial asymmetry (*n* = 1), and dysarthria (*n* = 2).

### 3.2. Laboratory examination results

All patients were positive for the MOG-IgG antibody. The number of leukocytes in the CSF samples of patients with MOGAD ranged between 2.0 × 10^6^ and 683.0 × 10^6^/L, with an average number of 63.3 × 10^6^/L. The number of leukocytes in patients with ADEM was significantly higher than that in patients with ON but lower than that in patients with TM (*p* < 0.05). The concentrations of CSF protein fluctuated between 84 and 1,307 mg/L, with a mean value of 295.8 mg/L, and no significant difference was detected among the groups. Among all patients, 3 (5.5%) were positive for EBV; 1 (1.9%) was positive for HSV type II; 9 (15.0%) were positive for serum EBV IgM antibody; and 14 (24.1%) were positive for serum *mycoplasma pneumoniae* IgM antibody (Table 1). The pathogen detection rate in this cohort was 36.5%. Of the three patients with EBV seropositivity, one exhibited fever but showed no signs of enlarged lymph nodes in the neck, enlarged liver or spleen, or abnormal liver function. There were also no abnormalities in the lymphocyte ratio and lymphocyte morphology. The other two patients did not present with any of these conditions.

**Table 1:** Laboratory examination results of patients with different clinical phenotypes of MOGAD.

	ADEM (*n* = 37)	Encephalitis (*n* = 11)	ON (*n* = 9)	NMOSD (*n* = 9)	TM (*n* = 6)	Leukodystrophy-like manifestations (*n* = 1)	Meningitis (*n* = 1)	TOTAL
Mean leukocyte count (×10^6^ L)	54.6	90.5	15.8	35.1	175.7	20	55	63.3
Mean protein concentration (mg/L)	286	241.9	193.1	290.8	502.1	280	465.8	295.8
EBV-positive in CSF (n of patients who tested positive/total n of patients tested)	1/27 (3.7%)	1/10 (10.0%)	0/6	0/7	1/5 (20.0%)	0/0	0/0	3/55 (5.5%)
HSV-positive in CSF (n of patients who tested positive/total n of patients tested)	0/24	1/10 (10.0%)	0/6	0/7	0/5	0/0	0/0	1/52 (1.9%)
EBV IgM-positive (n of patients who tested positive/total n of patients tested)	3/26 (11.5%)	3/10 (30.0%)	1/9 (11.1%)	1/8 (12.5%)	1/5 (20.0%)	0/1	0/1	9/60 (15.0%)
MP IgM-positive (n of patients who tested positive/total n of patients tested)	7/25 (28.0%)	1/10 (10.0%)	4/9 (44.4%)	1/8 (12.5%)	1/5 (20.0%)	0/0	0/1	14/58 (24.1%)

MOGAD, myelin oligodendrocyte glycoprotein antibody-associated disease; ADEM, acute disseminated encephalomyelitis; ON, optic neuritis; NMOSD, neuromyelitis optica spectrum disorder; TM, transverse myelitis; CSF, Cerebrospinal fluid; EBV, Epstein–Barr virus; HSV, herpes simplex virus; MP, mycoplasma pneumoniae.

### 3.3. Imaging characteristics

**Figure 4:**
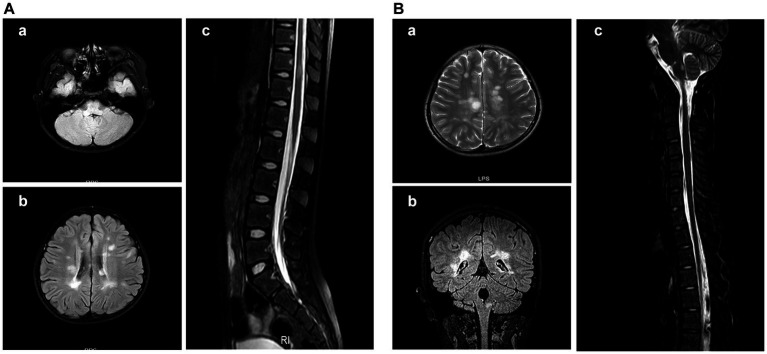
Cranial MRI results of the patient with area postrema syndrome. A female child, aged 133 months, presented with bouts of vomiting and dizziness for over 10 days. **(A)** Cranial MRI scan at admission. **(A-a)** Abnormal signal in the right side of the medulla oblongata in the FLAIR sequence. **(A-b)** Abnormal signal in the bilateral periventricular white matter in the FLAIR sequence. **(A-c)** Patchy abnormal signal at the T11 level in the FLAIR sequence. **(B)** Cranial MRI at 30 months after the first MRI scan. The patient complained about headache and had vomited for 2 days. **(B-a)** The periventricular semioval center of the lateral ventricle in the FLAIR sequence. **(B-b)** The right medulla oblongata and periventricular white matter in the coronal section of the FLAIR sequence. **(B-c)** Abnormal signal in the dorsal side of the medulla oblongata in the FLAIR sequence.

**Figure 5:**
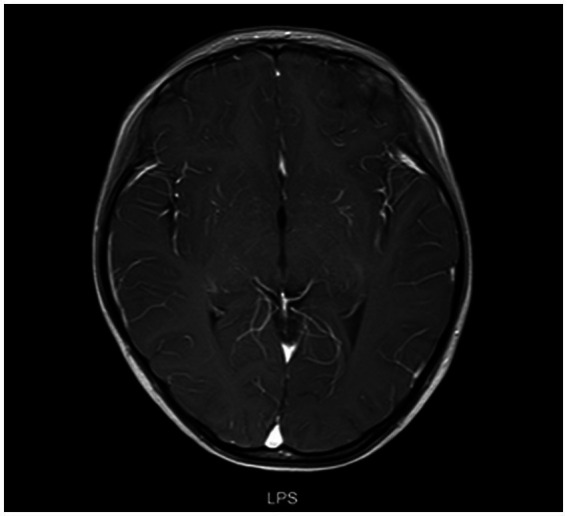
Cranial MRI results of a patient with meningitis who presented with fever. A female child, aged 78 months, was admitted to the hospital with fever for 9 days. At 19 days after admission, cranial enhancement MRI showed that the meninges of the right temporal pole were enhanced and no abnormality was found in the brain parenchyma.

All patients underwent MRI scans of the brain and spine. In addition to typical imaging manifestations of ADEM, NMOSD, ON, and TM, one patient with NMOSD presented with area postrema syndrome (Figure 4). Of three children who presented with encephalitis but exhibited no significant abnormalities on cranial MRI in the early stage of the disease, two showed thalamic lesions at 2 weeks after onset, while one exhibited no abnormalities throughout the disease course. One patient who had meningitis and presented with fever as the only clinical manifestation at admission exhibited meningeal enhancement on repeated cranial MRI (Figure 5).

The brain MRI scans indicated involvement in various locations, including the frontal lobe (*n* = 35), parietal lobe (*n* = 35), temporal lobe (*n* = 21), occipital lobe (*n* = 21), basal ganglia (*n* = 21), thalamus (*n* = 25), brainstem (*n* = 11), cerebellum (*n* = 15), corpus callosum (*n* = 8), and medulla oblongata (*n* = 4), as well as bilateral involvement of the optic nerve (*n* = 11) and unilateral involvement of the optic nerve (*n* = 3). The spinal cord MRI scans revealed affected areas in the cervical cord (*n* = 19), thoracic cord (*n* = 21), lumbar and sacral cord (*n* = 14), and long segment (*n* = 20).

### 3.4. Treatments

The shortest mean duration from admission to methylprednisolone therapy was observed in patients with ON and NMOSD (1.22 days and 1.44 days, respectively). The longest mean duration was found in patient with meningitis (19 days), followed by patients with encephalitis (6.54 days). Significant differences in the mean duration from admission to methylprednisolone therapy were observed in patients with NMOSD versus ADEM (*p* < 0.05), NMOSD versus encephalitis (*p* < 0.05), ON versus ADEM (*p* < 0.05), ON versus encephalitis (*p* < 0.05), and ADEM versus encephalitis (*p* < 0.05).

Of 74 patients, 15 (20.3%) received immunosuppressive therapy, including 8 patients with ADEM, 5 with NMOSD, 1 with ON, and 1 with TM. Patients were treated monthly with azathioprine (*n* = 7), mycophenolate mofetil (*n* = 5), cyclophosphamide (*n* = 2), and intravenous drip of gamma globulin (*n* = 2). One patient already taking azathioprine was treated with mycophenolate mofetil due to abnormal liver function. One patient was treated with mycophenolate mofetil due to the poor effect of azathioprine. Recurrence occurred in 13 patients (Table 2).

**Table 2:** Treatments received by patients with different clinical phenotypes of MOGAD.

	ADEM (*n* = 37)	Encephalitis (*n* = 11)	ON (*n* = 9)	NMOSD (*n* = 9)	TM (*n* = 6)	Leukodystrophy-like manifestations (*n* = 1)	Meningitis (*n* = 1)
Mean duration from admission to methylprednisolone therapy (days)	3.32	6.54	1.22	1.44	3.33	1	19
Mean duration of methylprednisolone therapy (days)	2.22	2.17	1.78	2.11	1.45	3	2
Gamma globulin, n	21	5	5	7	4	0	1
Gamma globulin (maintenance period), n	2	0	0	0	0	0	0
Azathioprine, n	3	0	1	2	1	0	0
Mycophenolate mofetil, n	3	0	1	1	0	0	0
Cyclophosphamide, n	0	0	0	2	0	0	0
Recurrence rate	8/37 (21.6%)	2/11 (18.2%)	2/9 (22.2%)	3/9 (33.3%)	2/6 (33.3%)	0/1	0/1

MOGAD, myelin oligodendrocyte glycoprotein antibody-associated disease; ADEM, acute disseminated encephalomyelitis; ON, optic neuritis; NMOSD, neuromyelitis optica spectrum disorder; TM, transverse myelitis.

### 3.5. Follow-up

**Figure 6:**
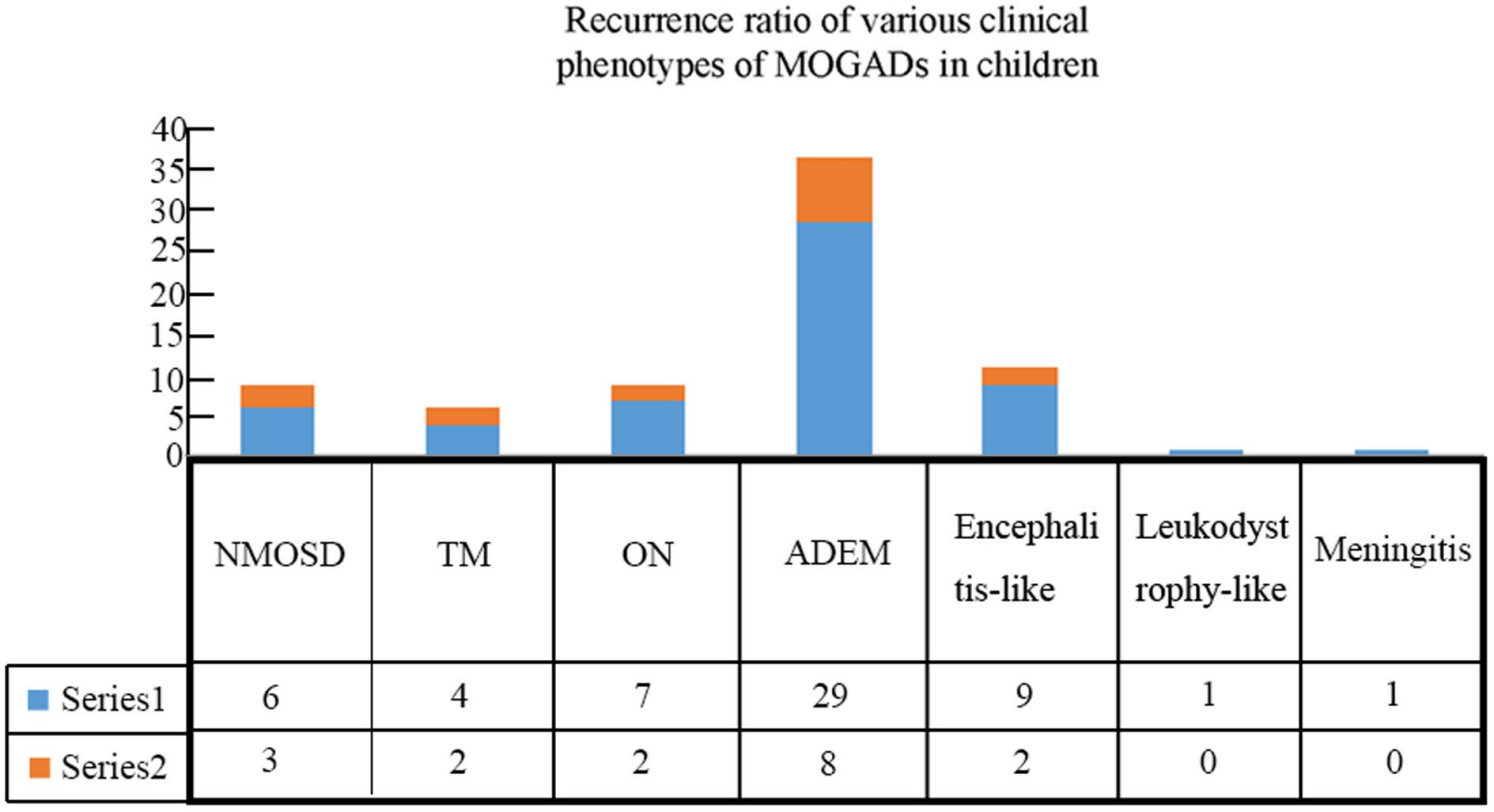
Recurrence rates among children with different clinical phenotypes of MOGAD.

Patients were followed for an average of 11.7 months (range, 60 days to 36 months). During follow-up, recurrence occurred in 17 (23.0%) children (Table 3), including 8 cases with ADEM (6 with multiphasic disseminated encephalomyelitis and 2 with ADEM-NMOSD), 2 with encephalitis (1 with NMOSD and 1 with seizures), 2 with ON (1 with ON-ADEM and 1 with ON-NMOSD), 3 with NMOSD, and 2 with TM (1 with TM-NMOSD and 1 with TM) (Figure 6). Among them, 6 experienced recurrence twice. The clinical features and MRI results of the different MOGAD syndromes are summarized in Supplementary Tables 1, 2, respectively. Representative MR images from patients with MDEM, relapsing NMOSD, TM-NMOSD, and encephalitis are shown in Supplementary Figures 1–4.

**Table 3:** Details of recurrence among pediatric patients with different clinical phenotypes of MOGAD.

	Age (months)	Sex	First phenotype	Treatment	Recurrence interval (months)	Phenotype of recurrence	Treatment	Immunosuppressive agents	Number of recurrences
1	138	F	ADEM	Steroids	7	ADEM	Oral Steroids	MMF	2
2	90	F	ADEM	Steroids	3, 5, 6	ADEM ADEM ADEM	Steroids pulse, IVIG	AZA MMF	3
3	55	F	ADEM	Steroids, IVIG	14	ADEM	Oral Steroids	MMF	1
4	105	F	ADEM	Steroids	24, 8	ADEM NMOSD	Steroids IVIG	No	2
5	59	F	ADEM	Steroids	10, 8	ADEM ADEM	Steroids	AZA	2
6	115	F	ADEM	Steroids IVIG	5, 3	ADEM ADEM	Steroids pulse	Immunoglobulin (6 times)	2
7	135	F	ADEM	Steroids	9	ADEM	Oral Steroids	Immunoglobulin (3 times)	1
8	107	F	ADEM	Steroids	6	ADEM	Oral Steroids	MMF	1
9	41	F	Encephalitis	Steroids IVIG	8	NMOSD	Steroids IVIG	No	1
10	150	M	Encephalitis	Steroids IVIG	3, 9, 11	EP	Steroids IVIG	No	3
11	117	M	ON	Steroids IVIG	7	ADEM	Steroids	MMF	1
12	109	M	ON	Oral Steroids	5	NMOSD	Steroids IVIG	AZA (withdrawal) MMF	1
13	85	F	NMOSD	Steroids IVIG	8	NMOSD	Steroids IVIG	Cyclophosphamide	1
14	140	F	NMOSD	Steroids IVIG	29	NMOSD	Steroids IVIG	Cyclophosphamide	1
15	172	F	NMOSD	Steroids IVIG	10	NMOSD	Steroids IVIG	AZA	2
16	95	F	TM	Steroids IVIG	5	NMOSD	Steroids	AZA	1
17	157	F	TM	Steroids	14	TM	Steroids	No	1

MOGAD, myelin oligodendrocyte glycoprotein antibody-associated disease; ADEM, acute disseminated encephalomyelitis; ON, optic neuritis; NMOSD, neuromyelitis optica spectrum disorder; TM, transverse myelitis; IVIG, intravenous immunoglobulins; EP, epilepsy; MMF, mycophenolate mofetil; AZA, Azathioprine.

Patient #3 exhibited multiphasic ADEM with spinal cord involvement. Patient #4 showed a clinical presentation of NMOSD with brain lesions.

The median age of patients with recurrence was 109 months, which was significantly higher than that of patients with no recurrence (82.5 months) (*p* < 0.05). The male-to-female ratio in patients with recurrence was 1:4.67, which was significantly lower than that in patients at first onset (*p* < 0.05). We further divided 74 patients into four groups according to their serum MOG levels: 1:10, 1:32, 1:100, and 1:320. Pairwise comparison analysis showed no significant difference in the recurrence rate among the four groups (*p* < 0.05) (Table 4). The average interval to the first recurrence was 9.8 months. All patients with recurrence were re-treated with hormone or immunosuppressive therapy, and the symptoms were relieved in the acute phase. Thirteen patients with recurrence were given immunosuppressants. Of them, a second recurrence occurred in 4 (30.8%) patients after treatment.

**Table 4:** Recurrence rates among patients with different serum MOG titers.

		Patients with recurrence (*n*)/total patients (*n*)
Serum MOG titer	1:10	9/39
	1:32	4/17
	1:100	2/13
	1:320	2/5

MOG, myelin oligodendrocyte glycoprotein.

Patient #10 experienced recurrent seizures characterized by frequent and intense episodes, which were controlled after immunomodulatory treatment, with a seizure-free period lasting several months. The main manifestation of the recurrence was seizures without fever or encephalopathy. Multiple cranial MRI showed no notable abnormalities. The initial presentation of this patient was fever and convulsions. The CSF white blood cell (WBC) count was 240 × 10^6^/L, with 64% monocytes. Cranial MRI did not reveal any significant abnormalities, but the electroencephalogram (EEG) indicated diffuse δ activity. The treatment consisted of methylprednisolone, intravenous immunoglobulin infusion, and oral sodium valproate. At the follow-up examination, the CSF WBC count was 32 × 10^6^/L, with 90% monocytes. The first follow-up examination was conducted after a 3-month interval, and the patient experienced recurrent seizures and status epilepticus again, without fever or signs of encephalopathy. The CSF WBC count was 36 × 10^6^/L, and the EEG showed increased δ activity in the left hemisphere and epileptic discharges in the left frontal lobe. Cranial and spinal MRI showed no abnormalities. The patient was treated with methylprednisolone, in addition to combination therapy with oxcarbazepine and sodium valproate to control the seizures. Subsequently, in the fourth month, occasional seizures occurred, and the CSF WBC count was 4 × 10^6^/L. The EEG indicated a basic rhythm with slow waves (≤8 Hz), and increased θ and δ activity was observed in the left hemisphere. Lacosamide was added to the treatment regimen for anti-seizure therapy, and seizure control was achieved. The second recurrence occurred 9 months after the previous one, with seizures again, but without fever or signs of encephalopathy. The CSF WBC count was 45 × 10^6^/L, and the EEG showed a basic rhythm with slow waves (≤8 Hz), as well as increased θ and δ activity in the left hemisphere. Cranial MRI was normal. The treatment included methylprednisolone, intravenous immunoglobulin infusion, and mycophenolate mofetil as immunomodulatory therapy, in addition to continued oral sodium valproate, oxcarbazepine, and lacosamide for anti-seizure therapy. Seizure control was achieved. The third recurrence occurred 11 months after the previous one, with seizures again, but without fever or signs of encephalopathy. The CSF WBC count was 97 × 10^6^/L, and the EEG showed a basic rhythm with slow waves (≤8 Hz), as well as increased θ and δ activity in the left hemisphere. Cranial MRI was normal. Methylprednisolone, mycophenolate mofetil, sodium valproate, oxcarbazepine, and lacosamide were used for anti-seizure therapy. The child remained seizure-free for nearly 1 year.

## 4. Discussion

Pediatric patients with MOGAD exhibit a wide range of monophasic and recurrent disorders (10). In this study, the most common clinical phenotype of MOGAD was ADEM, accounting for 50% of the children. This finding is consistent with the study reported by the E.U. Pediatric MOG Consortium, which showed that 46% of pediatric patients with MOGAD presented with ADEM (11). The second most common phenotype was encephalitis (14.9%), with a higher prevalence rate in this cohort than in the above-mentioned E.U. study (11), which may be explained by a higher detection rate of the MOG antibody in our study. Thus, to achieve early diagnosis, testing for both autoimmune encephalitis antibodies and CNS demyelinating antibodies should be conducted in children with encephalitis. Patients with TM had the highest median age of disease onset compared to those with other clinical phenotypes, suggesting that elder children were prone to have lesions in the spinal cord. The shortest hospital stay was observed in patients with ON, possibly because steroid pulse therapy was administered to them immediately after admission. Children with meningitis had the longest hospital stay, which may be related to the lack of neurological signs and symptoms, which leads to the late diagnosis of these patients.

The CSF leukocyte count was elevated in all clinical phenotypes of MOGAD in comparison with the healthy range, while no significant increase in the CSF protein concentration was observed. Atypical changes in imaging examinations are often observed in patients with MOGAD. In a previous cohort study of 56 children positive for MOG antibodies, 8 (14%) exhibited symptoms of area postrema syndrome (APS), with 7 presenting both ADEM and APS, and 1 displaying APS alone (12). In a multicenter study of 274 adult patients with positive AQP4 antibodies and 107 adult patients positive for MOG antibodies in South Korea, 41 (14.9%) AQP4-positive patients had the first symptom of APS, and 47 (17.2%) exhibited APS during the disease, while no patient positive for MOG antibodies had the first symptoms of APS, and only 2 (1.9%) patients exhibited APS symptoms during the disease (13). Venkatesan et al. (14) found that 9% of patients with MOG-related encephalitis phenotype showed normal cranial MRI results (8), which was consistent with our study, in which 1 out of 11 (9.1%) patients with encephalitis exhibited no cranial MRI abnormalities throughout the disease course. The above data suggest that patients with MRI-negative encephalitis may have MOGAD and should be observed for demyelination during follow-up. The cranial MRI scans of a meningitis patient with fever as the only clinical manifestation showed meningeal enhancement without parenchymal changes, suggesting consistent absence of demyelinating changes in this patient.

The incidence of EBV positivity in other patients, such as those with fever, was much lower compared to that in patients with MOGAD. Previous studies have indicated a potential link between viral infection and the development of MOGAD (15, 16). Despite the absence of evidence for intracellular EBV infection in these specific patients, we believe it is important to consider the possibility of EBV infection being associated with MOGAD and to further investigate this correlation. In addition, patient #10 experienced recurrent seizures. Although the cranial MRI did not show any notable abnormalities, there was a decrease in the cerebrospinal fluid leukocyte count, which later increased. In addition, the serum MOG antibody titers at a dilution of 1:10 were positive in multiple tests, and the seizure symptoms were improved following immunomodulatory therapy. MOG encephalitis occurred 22 days after HSV encephalitis. Therefore, we considered that the seizures were associated with MOG positivity.

Methylprednisolone pulse therapy and immunoglobulin immunomodulatory therapy are efficient first-line treatments for MOGAD, especially in the acute phase (17). In this study, all patients received methylprednisolone pulse therapy and 43 (58.1%) also were given concomitant immunomodulatory therapy with gamma globulin. Treatment with methylprednisolone pulse therapy is associated with significantly decreased visual acuity, reduced risk of disability, and improved early diagnosis in patients with ON and NMOSD. Patients with encephalitis received methylprednisolone pulse therapy after 6.54 days of admission, which was significantly different compared to patients with NMOSD, ON, and ADEM. Therefore, the recognition of MOGAD patients with encephalitis needs to be improved. Among 15 patients who were given immunosuppressive therapy, there were 2 with monophasic disease. Both patients were diagnosed with the NMOSD phenotype, presenting with acute onset and significant visual impairment. Despite two courses of high-dose steroid therapy and intravenous immunoglobulin treatment, they experienced limited improvement in visual acuity. Considering the potentially disabling nature of the disease and the anticipated long-term use of steroids, immunosuppressive therapy was prescribed. The highest recurrence rates in this study were observed in patients with NMOSD (33%) and TM (33%), which is consistent with the findings of Waters et al. (18), who showed that the recurrence of MOGAD was associated with persistent positive antibodies and patients who remained seropositive during follow-up had a higher risk of recurrence than those who converted to seronegative status (38% vs. 13%). In the present study, we found no association between high serum MOG antibody titers at the beginning of the disease course and the rate of disease recurrence.

## 5. Limitations

The sample size for each clinical phenotype was small, and all patients were from the same hospital. Additionally, the follow-up period was short for some patients. Future investigations with a larger sample size and longer follow-up are needed.

## 6. Conclusion

This study showed that ADEM and encephalitis are the most common clinical phenotypes of MOGAD in Chinese pediatric patients. The age of onset, hospital stay, number of leukocytes in the CSF, and recurrence rate significantly differed in children with different MOGAD phenotypes. Moreover, atypical MRI changes were found in some MOGAD cases. These findings contribute to a better understanding of the characteristics, treatment options, and recurrence rates of each clinical phenotype of MOGAD and may provide insight for improving the diagnosis and treatment of these diseases.

## Data availability statement

The raw data supporting the conclusions of this article will be made available by the authors, without undue reservation.

## Ethics statement

The studies involving humans were approved by the Department of Neurology of Children’s Hospital Affiliated to Zhejiang University School of Medicine. The studies were conducted in accordance with the local legislation and institutional requirements. Written informed consent for participation in this study was provided by the participants’ legal guardians/next of kin.

## Author contributions

YH wrote the main manuscript text. XY prepared Tables 1–4. LL prepared Figures 1–6. YW and LX statistics. PJ and ZY specialist in neurology. FG concept and design. All authors reviewed the manuscript and approved the final draft before submission.

## Funding

This study is supported by Key Research and Development Plan of Zhejiang Province (2020C03038); National Natural Science Foundation of China (Grant No. 82001232); Zhejiang Provincial Natural Science Foundation of China (Grant No. LQ19H090002); STI2030-Major Projects+2021ZD 0201700.

## Conflict of interest

The authors declare that the research was conducted in the absence of any commercial or financial relationships that could be construed as a potential conflict of interest.

## Publisher’s note

All claims expressed in this article are solely those of the authors and do not necessarily represent those of their affiliated organizations, or those of the publisher, the editors and the reviewers. Any product that may be evaluated in this article, or claim that may be made by its manufacturer, is not guaranteed or endorsed by the publisher.
