# Supplementary material for: Phenotypic characteristics of myelin oligodendrocyte glycoprotein antibody-associated disease in children: a single-center, retrospective study
Source: Front Neurol. 2023 Aug 29;14:1188323. doi: 10.3389/fneur.2023.1188323 (PMC10495833; doi:10.3389/fneur.2023.1188323)
Supplement: Supplementary file 1 [file Data_Sheet_1.pdf]

Supplementary Table 1. Clinical features of each MOGAD syndrome

MOGAD syndrome	Clinical features
MDEM	Multiple episodes of acute-onset diffuse brain parenchymal involvement with meningeal and spinal cord inflammation, increased mononuclear cells in the cerebrospinal fluid, widespread moderate abnormalities in electroencephalogram, and multiple scattered lesions in the brain and spinal cord on CT or MRI. Relapse characterized by the appearance of clinical symptoms and new lesions on radiological scans at least 1 month after the last acute attack.
ADEM-ON	Patients initially present with ADEM and subsequently experience recurrent demyelinating episodes limited to the optic nerves.
Relapsing NMOSD	Multiple episodes with typical features of NMOSD: ON, acute myelitis, area postrema syndrome, acute brainstem syndrome, acute diencephalic syndrome, and cerebral syndrome. Patients initially present with an NMOSD-like phenotype and may experience subsequent relapses, primarily involving ON or both ON and TM.
ON-NMOSD or TM-NMOSD	Patients initially present with isolated ON or TM and may experience subsequent relapses, transforming into a relapsing NMOSD-like phenotype during follow-up.
ADEM-NMOSD	Patients initially present with ADEM and may experience subsequent transition to NMOSD due to further relapses, such as simultaneous or sequential occurrences of ON or TM.
Encephalitis	Mainly characterized by encephalopathy, including impaired consciousness, seizures, fever, behavioral abnormalities, and movement disorders. Brain MRI shows involvement of the cerebral cortex or no abnormalities.
Epilepsy	Isolated seizure episodes that may be followed by central nervous system demyelinating events months or years later.

Supplementary Table 2. MRI findings for each MOGAD syndrome

MOGAD syndrome	MRI findings
MDEM	Brain MRI consistently shows disseminated encephalomyelitis, including asymmetric distribution of high-signal lesions in the cerebral white matter and relatively symmetric involvement of deep gray matter structures (such as the thalamus and basal ganglia). Lesions typically have indistinct borders and are commonly found in the subcortical and deep white matter regions, less frequently involving the corpus callosum.<br>(**Supplementary Figure 1A-F**)
ADEM-ON	During the initial episode, brain MRI findings are consistent with ADEM, whereas in subsequent relapses, the MRI findings match those of ON. It is characterized by either normal brain MRI or non-specific white matter lesions. Lesions typically involve more than half the length of the optic nerve. During the acute phase, the optic nerve is thickened with enhancement, sometimes accompanied by enhancement of surrounding tissues. In the remission phase, the optic nerve shows atrophy and thinning, leading to the formation of double-track signs, although it can also be negative.
Relapsing NMOSD	In multiple episodes, brain MRI findings consistently match the characteristics of NMOSD:<br>1. Acute ON: Normal brain MRI or non-specific white matter lesions; long T2 signal or T1 enhancement involving at least half the length of the optic nerve or involvement of the optic chiasm.<br>2. Acute myelitis: Longitudinally extensive spinal cord lesions involving at least three contiguous vertebral segments, or corresponding spinal cord atrophy in patients with a history of myelitis involving at least three contiguous vertebral segments.<br>3. Area postrema syndrome: Lesions in the dorsal medulla/area postrema.<br>4. Acute brainstem syndrome: Periependymal lesions around the brainstem aqueduct. New lesions typically correlate with new symptoms, with an interval usually >3 months.<br>(**Supplementary Figure 2A-D**)
ON-NMOSD or TM-NMOSD	Initial brain MRI findings are consistent with either ON or TM. In subsequent relapses, MRI findings are consistent with NMOSD.<br>(**Supplementary Figure 3A-D**)
ADEM-NMOSD	Initial brain MRI findings match those of ADEM, while in subsequent relapses, the MRI findings are consistent with NMOSD.
Encephalitis	Brain MRI shows primarily cortical involvement, with widespread and bilateral cortical lesions in most patients. Some patients may have additional involvement of isolated basal ganglia or thalamic structures. In a minority of cases, head MRI shows no abnormalities.

	(**Supplementary Figure 4A, B**)
Epilepsy	Initial MRI findings may not necessarily be abnormal, but months or years later, MRI reveals central nervous system demyelinating lesions.

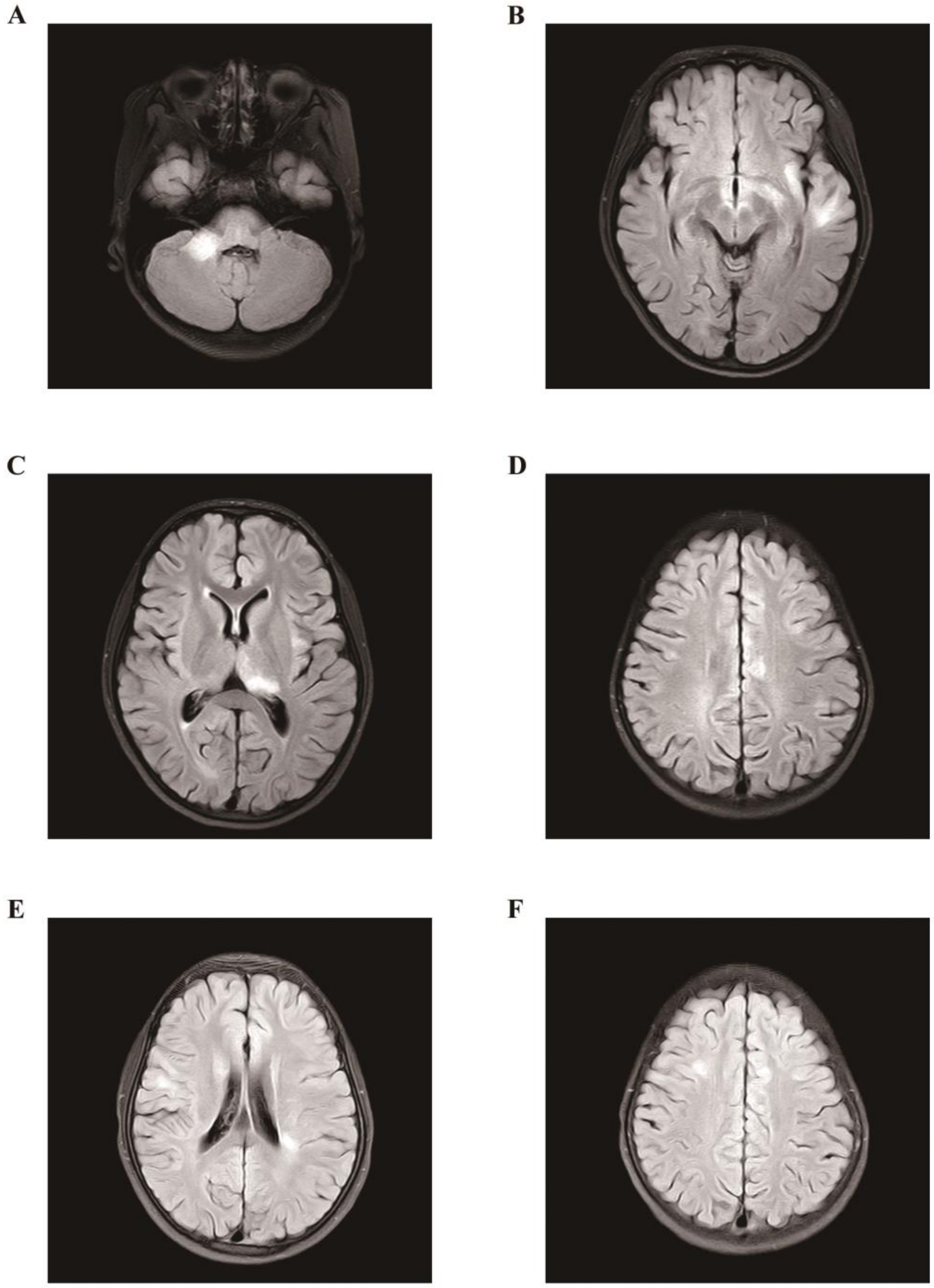

Supplementary Figure 1. Representative MR images from a patient with MDEM (Patient #2)

(A-D) Representative MR images captured during the initial event. (A) Abnormal signal in the right cerebellum. (B) Abnormal signal in bilateral basal ganglia area and left temporal lobe. (C) Abnormal signal in left thalamus. (D) Abnormal signal in bilateral parietal lobes and semioval center. (E-F) Representative MR images captured during the relapsing event. (E) Abnormal signal in the right frontoparietal lobe and bilateral paraventricular. (F) Abnormal signal in bilateral frontal lobe.

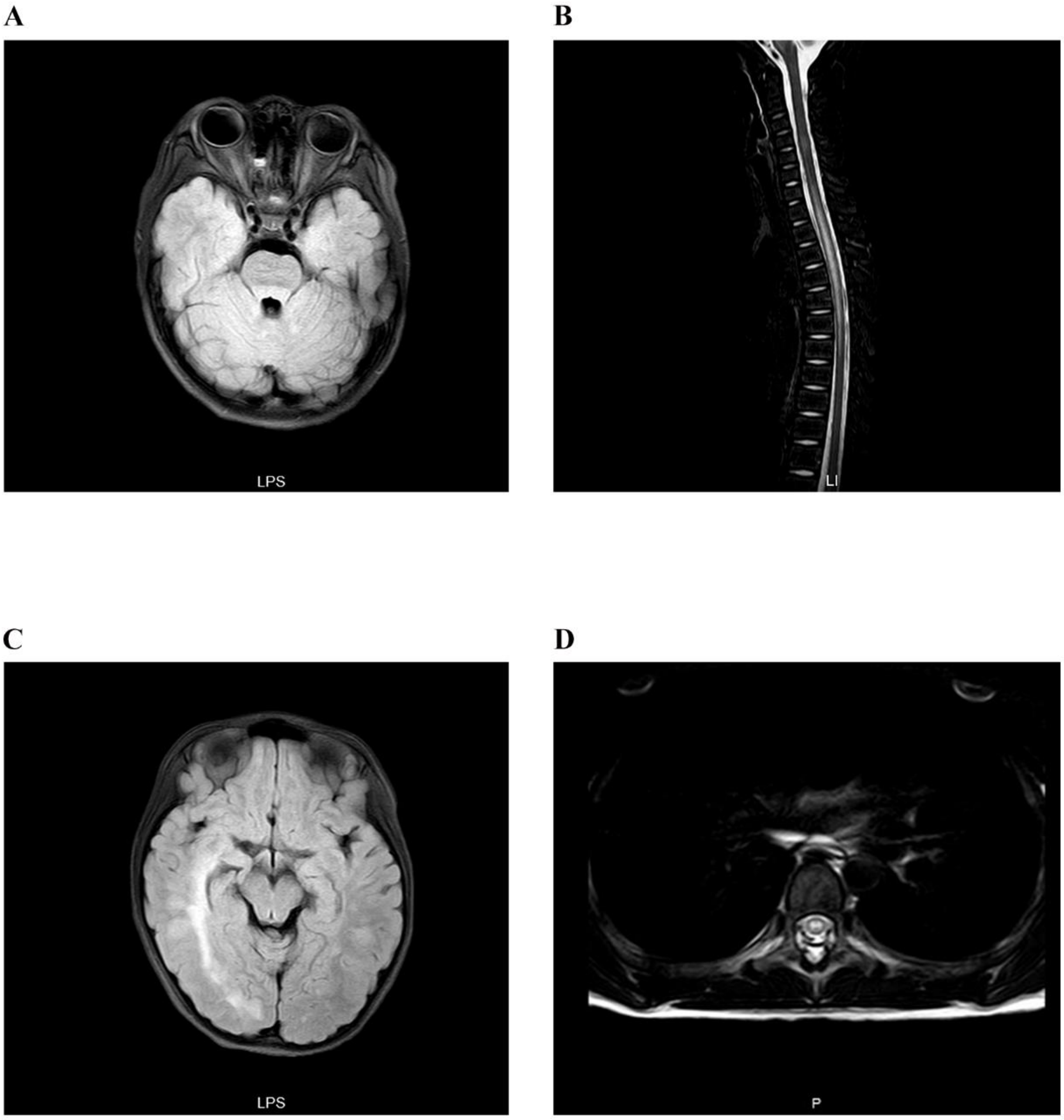

Supplementary Figure 2. Representative MR images from a patient with relapsing NMOSD (Patient #13)

(A) Abnormal signal in bilateral optic nerves. (B) Abnormal signal in the long segment of the spinal cord C5-T9. (C) Abnormal signal in the white matter of the right temporal

and occipital lobes. (D) Abnormal signal in the transverse position of the spinal cord ("H" sign).

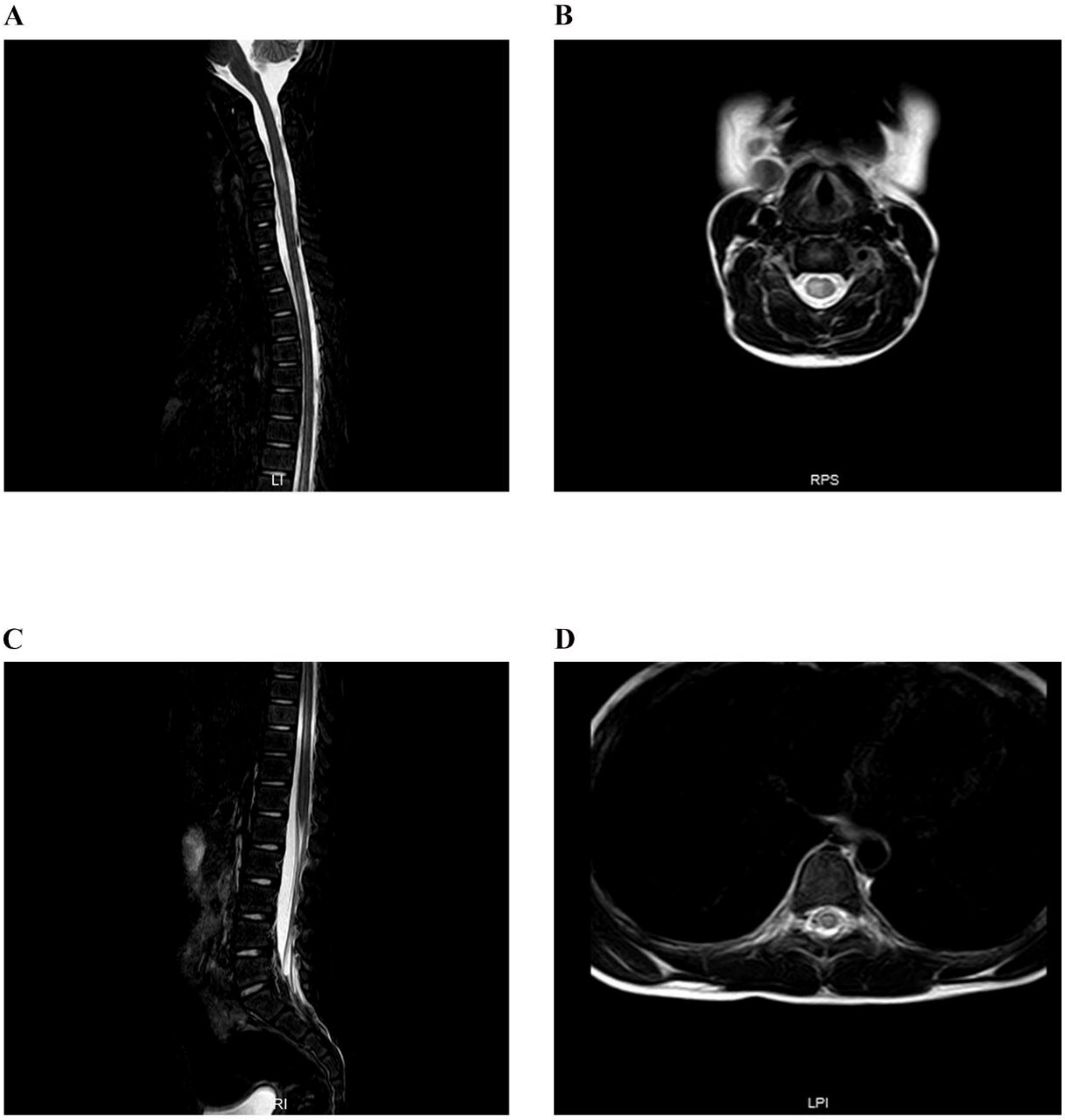

Supplementary Figure 3. Representative MR images from a patient with TM-NMOSD (Patient #16)

(A, B) Abnormal signals in the long segment of the spinal cord C5-T10. (C, D) Abnormal signal in the transverse spinal cord.

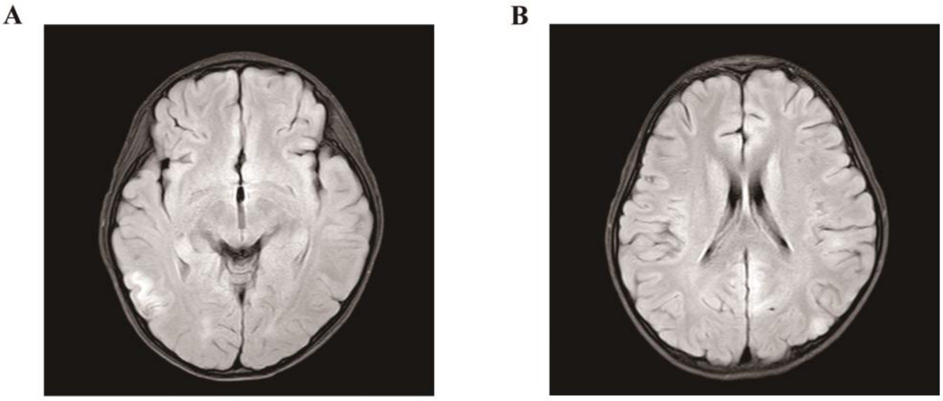

Supplementary Figure 4. Representative MR images from a patient with encephalitis (Patient #65)

(A) Abnormal signal in the right temporal and occipital cortex. (B) Abnormal signal in the left occipital cortex.
